# Correction: Can self-efficacy mediate between knowledge of policy, school support and teacher attitudes towards inclusive education?

**DOI:** 10.1371/journal.pone.0262625

**Published:** 2022-01-11

**Authors:** Shirli Werner, Thomas P. Gumpel, Judah Koller, Vered Wiesenthal, Naomi Weintraub

The second author’s name is spelled incorrectly. The correct name is: Thomas P. Gumpel.
