# Supplementary material for: Can self-efficacy mediate between knowledge of policy, school support and teacher attitudes towards inclusive education?
Source: PLoS One. 2021 Sep 20;16(9):e0257657. doi: 10.1371/journal.pone.0257657 (PMC8452075; doi:10.1371/journal.pone.0257657)
Supplement: Attachment — Submitted filename: PONE-D-20-30355_reviewer with comments.pdf [file pone.0257657.s002.pdf]

PLOS ONE

Can self-efficacy mediate between knowledge of policy, school support and teachers' attitudes towards inclusive education?

--Manuscript Draft--

**Manuscript Number:**	PONE-D-20-30355
**Article Type:**	Research Article
**Full Title:**	Can self-efficacy mediate between knowledge of policy, school support and teachers' attitudes towards inclusive education?
**Short Title:**	Teachers' attitudes towards inclusion education
**Corresponding Author:**	Shirli Werner, Ph.D.<br>Hebrew University of Jerusalem<br>Jerusalem, ISRAEL
**Keywords:**	school inclusion; inclusive education; special education; general education; attitudes; teachers; self-efficacy
**Abstract:**	Background: While research has focused on understanding teachers' attitudes towards the inclusion of children with special educational needs into general education classrooms, there are lacunae that have yet to be addressed. This study examined the association between perceived self-efficacy and attitudes towards inclusion among elementary school teachers. The study also examined the role of teachers' self-efficacy as a mediating variable between knowledge of inclusion policy, perception of school support and teachers' attitudes towards inclusion.<br>Methods: Teachers (N=352) working in general or special education schools completed questionnaires assessing attitudes towards inclusion, sense of self-efficacy, knowledge of current policy, and perception of support for inclusive practices.<br>Results: Higher perceived knowledge of inclusion policy and higher perceived school support of inclusion were both related to higher self-efficacy regarding inclusion, which, in turn, was related to more positive attitudes about inclusion.<br>Conclusion: Our results suggest that point to being knowledgeable regarding local and national policy is important in order to increase feelings of self-efficacy regarding the implementation of effective educational practice. To enhance inclusion, local and national policy must be clearly communicated to teachers. Furthermore, leadership and a supportive school environment are conducive to successful inclusive education.
**Order of Authors:**	Shirli Werner, Ph.D.<br>Thomas P Gumpel<br>Judah Koller<br>Vered Wiesenthal<br>Naomi Weintraub
**Additional Information:**	
**Question**	**Response**
**Financial Disclosure**<br><br>Enter a financial disclosure statement that describes the sources of funding for the work included in this submission. Review the submission guidelines for detailed requirements. View published research articles from *PLOS ONE* for specific examples.<br><br>This statement is required for submission	Funding for this study was provided by the Ministry of Education, Israel.

Powered by Editorial Manager® and ProduXion Manager® from Aries Systems Corporation

and **will appear in the published article** if the submission is accepted. Please make sure it is accurate.<br><br>**Unfunded studies**<br>Enter: *The author(s) received no specific funding for this work.*<br><br>**Funded studies**<br>Enter a statement with the following details:<br>• Initials of the authors who received each award<br>• Grant numbers awarded to each author<br>• The full name of each funder<br>• URL of each funder website<br>• Did the sponsors or funders play any role in the study design, data collection and analysis, decision to publish, or preparation of the manuscript?<br>• **NO** - Include this sentence at the end of your statement: *The funders had no role in study design, data collection and analysis, decision to publish, or preparation of the manuscript.*<br>• **YES** - Specify the role(s) played.<br><br>* typeset	
**Competing Interests**<br><br>Use the instructions below to enter a competing interest statement for this submission. On behalf of all authors, disclose any competing interests that could be perceived to bias this work—acknowledging all financial support and any other relevant financial or non-financial competing interests.<br><br>This statement **will appear in the published article** if the submission is accepted. Please make sure it is accurate. View published research articles from *PLOS ONE* for specific examples.	The authors have declare that no competing interests exist.

**NO authors have competing interests**<br><br>Enter: *The authors have declared that no competing interests exist.*<br><br>**Authors with competing interests**<br><br>Enter competing interest details beginning with this statement:<br><br>*I have read the journal's policy and the authors of this manuscript have the following competing interests: [insert competing interests here]*<br><br>* typeset	
**Ethics Statement**<br><br>Enter an ethics statement for this submission. This statement is required if the study involved:<br><br>• Human participants<br>• Human specimens or tissue<br>• Vertebrate animals or cephalopods<br>• Vertebrate embryos or tissues<br>• Field research<br><br>Write "N/A" if the submission does not require an ethics statement.<br><br>General guidance is provided below. Consult the submission guidelines for detailed instructions. **Make sure that all information entered here is included in the Methods section of the manuscript.**	This study was approved by the IRB of the Seymour Fox School of Education, Hebrew University of Jerusalem, Jerusalem, Israel

Powered by Editorial Manager® and ProduXion Manager® from Aries Systems Corporation

**Format for specific study types**<br><br>**Human Subject Research (involving human participants and/or tissue)**<br>• Give the name of the institutional review board or ethics committee that approved the study<br>• Include the approval number and/or a statement indicating approval of this research<br>• Indicate the form of consent obtained (written/oral) or the reason that consent was not obtained (e.g. the data were analyzed anonymously)<br><br>**Animal Research (involving vertebrate animals, embryos or tissues)**<br>• Provide the name of the Institutional Animal Care and Use Committee (IACUC) or other relevant ethics board that reviewed the study protocol, and indicate whether they approved this research or granted a formal waiver of ethical approval<br>• Include an approval number if one was obtained<br>• If the study involved *non-human primates*, add *additional details* about animal welfare and steps taken to ameliorate suffering<br>• If anesthesia, euthanasia, or any kind of animal sacrifice is part of the study, include briefly which substances and/or methods were applied<br><br>**Field Research**<br><br>Include the following details if this study involves the collection of plant, animal, or other materials from a natural setting:<br>• Field permit number<br>• Name of the institution or relevant body that granted permission	
**Data Availability**<br><br>Authors are required to make all data underlying the findings described fully available, without restriction, and from the time of publication. PLOS allows rare exceptions to address legal and ethical concerns. See the PLOS Data Policy and FAQ for detailed information.	Yes - all data are fully available without restriction

A Data Availability Statement describing where the data can be found is required at submission. Your answers to this question constitute the Data Availability Statement and **will be published in the article**, if accepted.<br><br>**Important:** Stating 'data available on request from the author' is not sufficient. If your data are only available upon request, select 'No' for the first question and explain your exceptional situation in the text box.<br><br>Do the authors confirm that all data underlying the findings described in their manuscript are fully available without restriction?	
**Describe where the data may be found in full sentences. If you are copying our sample text, replace any instances of XXX with the appropriate details.**<br><br>• If the data are **held or will be held in a public repository**, include URLs, accession numbers or DOIs. If this information will only be available after acceptance, indicate this by ticking the box below. For example: *All XXX files are available from the XXX database (accession number(s) XXX, XXX.).*<br>• If the data are all contained **within the manuscript and/or Supporting Information files**, enter the following: *All relevant data are within the manuscript and its Supporting Information files.*<br>• If neither of these applies but you are able to provide **details of access elsewhere**, with or without limitations, please do so. For example:<br><br>*Data cannot be shared publicly because of [XXX]. Data are available from the XXX Institutional Data Access / Ethics Committee (contact via XXX) for researchers who meet the criteria for access to confidential data.*<br><br>*The data underlying the results presented in the study are available from (include the name of the third party*	The data underlying the results presented in the study are available from the first author via e-mail.

and contact information or URL).<br>• This text is appropriate if the data are owned by a third party and authors do not have permission to share the data.<br><br>* typeset	
Additional data availability information:	Tick here if your circumstances are not covered by the questions above and you need the journal’s help to make your data available.

Cover Letter
Click here to access/download;Manuscript;Attitudes toward inclusion 25.9.docx

Can self-efficacy mediate between knowledge of policy, school support and teachers' attitudes towards inclusive education?

S. Werner[1], T. P. Gumpel [2], J. Koller [2], V. Wiesenthal [2], N. Weintraub [3]

1. Paul Baerwald School of Social Work and Social Welfare, Hebrew University of Jerusalem, Jerusalem, Israel

2. Seymour Fox School of Education, Hebrew University of Jerusalem, Jerusalem, Israel

3. Faculty of Medicine, School of Occupational Therapy, Hebrew University of Jerusalem, Jerusalem, Israel

Can self-efficacy mediate between knowledge of policy, school support and teachers' attitudes towards inclusive education?

Abstract

Background: While research has focused on understanding teachers’ attitudes towards the inclusion of children with special educational needs into general education classrooms, there are lacunae that have yet to be addressed. This study examined the association between perceived self-efficacy and attitudes towards inclusion among elementary school teachers. The study also examined the role of teachers' self-efficacy as a mediating variable between knowledge of inclusion policy, perception of school support and teachers' attitudes towards inclusion.

Methods: Teachers (N=352) working in general or special education schools completed questionnaires assessing attitudes towards inclusion, sense of self-efficacy, knowledge of current policy, and perception of support for inclusive practices.

Results: Higher perceived knowledge of inclusion policy and higher perceived school support of inclusion were both related to higher self-efficacy regarding inclusion, which, in turn, was related to more positive attitudes about inclusion.

Conclusion: Our results suggest that point to being knowledgeable regarding local and national policy is important in order to increase feelings of self-efficacy regarding the implementation of effective educational practice. To enhance inclusion, local and national policy must be clearly communicated to teachers. Furthermore, leadership and a supportive school environment are conducive to successful inclusive education.

Keywords: school inclusion, inclusive education, special education, general education, attitudes, teachers, self-efficacy

Introduction

School inclusion and teachers' attitudes

School inclusion refers to the acceptance of students with disabilities into general education classrooms while responding to their diverse needs and providing the intervention and support necessary for them to succeed [1]. While multiple variables are associated with the success of inclusion, teachers' attitudes towards inclusion, which is the focus of this article, are the key determinant [2].

Just as inclusion is a multi-faceted construct, attitudes regarding inclusionary practices are complex. Mahat [3] suggested that attitudes include cognitive, affective, and behavioral dimensions. The cognitive dimension reflects teachers' beliefs regarding inclusion, the affective dimension represents associated emotions, and the behavioral dimension reflects teachers' intention to act in a certain manner. Cullen et al. [4] suggested that attitudes are comprised of teachers' perceptions of students with disabilities within inclusive settings, beliefs about the efficacy of inclusion and perceptions of the teachers' own professional roles, functions, and abilities [4].

While teachers' attitudes towards inclusion have been examined in many studies and countries, conflicting results indicate the need for continued research. Some studies show that teachers acknowledge the importance of inclusion [5, 6]. For example, pre-service teachers in Australia held generally positive attitudes towards inclusion and these attitudes were associated with years of training [7]. Other studies show that some teachers hold negative attitudes toward inclusion [8], see the inclusive process as problematic [9] and favor a separate special education system [10]. Further, some teachers prefer to not be involved in inclusion [11] and perceive it to be too time consuming [12]. In Israel, teachers felt that inclusion, especially of students with

intellectual disabilities, failed due to insufficient resources provided to teachers and the limited professional abilities of general education teachers [11].

Students with special education needs are often initially educated in special education settings. The process of inclusion requires that teachers in special education schools make recommendations regarding the transition of these students into the general education system, while general education teachers are the main agents in implementing the inclusion policy [13]. Therefore, the attitudes of teachers in both systems are important. Studies comparing the attitudes of teachers in general and special education systems are scarce [5, 13, 14]. Most studies have compared teachers according to their role (regardless of work setting), and found more positive attitudes among special education teachers compared to general education teachers [5, 13, 14].

Teachers' self-efficacy, knowledge of policy, school support and attitudes towards inclusion

There are different factors which have been found to be associated with teachers' attitudes towards inclusion. One frequently examined factor in the context of attitudes towards inclusion is teachers' self-efficacy. Self-efficacy is a future-oriented belief relating to the individuals' confidence and perceived ability to perform a given behavior [15]. Teachers' self-efficacy relates to their confidence and perceived ability to provide academic instruction, create a positive learning environment, and influence how well their students learn [16, 17]. Teachers with low self-efficacy were found to be more likely to ascribe difficulties in learning to the child and less willing to adapt their teaching methods [18].

The literature examining the association between teacher self-efficacy and their attitudes towards inclusion is inconclusive. Some found a relationship between teacher' self-efficacy and successful inclusive teaching [17] or their attitudes toward

inclusion [19, 20, 21]. Others found a weak relationship between these variables [6, 10]. Still others indicate that only certain aspects of self-efficacy, such as efficacy in collaboration with other teachers and parents [22] or regarding instructional strategies [17], were related to attitudes towards inclusion.

Beyond self-efficacy, two additional variables are important in understanding attitudes towards inclusion: knowledge of inclusion policy and school support of inclusion. We contend that these two variables may have a potential direct association with attitudes towards inclusion, as well as an indirect association via their possible connection with self-efficacy. Previous studies that examined the association between knowledge, school support and inclusion have largely focused on inclusionary practices or implementation of inclusion as opposed to attitudes towards inclusion [e.g. 23, 24]. Since attitudes towards inclusion are a key determinant of inclusive practice [2], we maintain that the association found regarding inclusion implementation is also relevant to attitudes toward inclusion.

Teacher's knowledge regarding inclusion policy has been selected for this study based on studies showing that teachers' knowledge regarding praxis is an important predictor of successful implementation of inclusion [e.g. 23, 24, 25]. Previous studies examined teachers' knowledge and skills of inclusive practices or teachers' knowledge of disability and have found these to be related to attitudes towards inclusion [21]. Studies have also shown knowledge to be related to self-efficacy given that teachers who are knowledgeable feel they have skills and confidence in implementing inclusionary practices [14, 26].

In the current study we focus on a different aspect of knowledge, that of knowledge regarding inclusion policy. Examining knowledge regarding inclusion policy is significant because in order for teachers to successfully implement inclusion

in their school system, they must be familiar with relevant local and national policy. Further, examining this factor is in line with Weatherley and Lipsky's notion of "street-level bureaucrats" [27, p. 172], seeing the lower-level staff as having great importance in the policy-making process and suggesting that the meaning of policy must be clear and transparent at the street level. Thus, we examined the association between teachers' knowledge of inclusion policy and their attitudes towards inclusion as well as how this relationship may be mediated by teachers' perceived self-efficacy.

School support of inclusion was selected for this study based on studies stressing the vital importance of school leadership and receiving support within the educational system in which they work [28]. Specifically, school climate, support from school leadership (e.g., administrators), relationships among teachers and the attitudes of other teachers are important factors in successful inclusion [17, 29]. Beyond its association with attitudes towards inclusion, the principal's leadership and relationships between teachers appear to be related to teacher's professional self-efficacy. Teachers who believe they work in a positive and supportive school environment are more likely to perceive themselves as capable of teaching students with disabilities [17]. Thus, teachers' perceived self-efficacy may mediate between school support of inclusion and attitudes towards inclusion.

Demographic and occupational predictors

Several demographic and occupational variables have been noted to be relevant to understanding teachers' attitudes towards inclusion. Some research has indicated that female teachers typically hold more positive attitudes towards inclusion than males [6, 7, 10, 30, 31], while others found no gender-based differences [2, 32, 33]. Some research points to teachers' age being unrelated to attitudes towards

inclusion [34], while other studies show that younger teachers have more positive attitudes [6, 10, 31, 35].

Regarding training and experience, again, the literature paints a conflicting picture. Some studies report that higher levels of education and fewer years of teaching experience were associated with more positive attitudes towards inclusion [19, 36]. In contrast, Yadav et al. [33] found that teachers with fewer than ten years of experience expressed most concerns about inclusion. One consistent finding points to the fact that training in special and inclusive education is correlated with positive attitudes towards inclusion [2, 5, 31, 36, 37, 38, 39].

Aims of the current study

While much attention has been paid to understanding teachers' attitudes towards inclusion, there are relative lacunae that have yet to be addressed. First, most studies have not compared attitudes towards inclusion between teachers who work in general versus special education settings. Second, while some studies have examined the role of knowledge regarding inclusion practice, they did not specifically focus on knowledge of relevant policy. Third, we could find no studies examining the mediating role of teachers' professional self-efficacy in implementing inclusionary practices, in the relationship between knowledge of inclusionary policy, perception of school support and attitudes towards inclusion.

The aims of the current study are therefore threefold. First, to examine the association between self-efficacy and attitudes towards inclusion among Israeli elementary school teachers who worked in general and special education schools. Second, to examine differences in attitudes towards inclusion between teachers working in general versus special education settings. Third, to examine the role of general education teachers' self-efficacy as a mediating variable between knowledge

of inclusion policy, perception of school support and teacher attitudes towards inclusion.

Specifically, we examined the following hypotheses:

1. Associations will be found between background (demographic, educational, and occupational) variables and attitudes toward inclusion. Teachers with a higher academic degree, fewer years of experience, homeroom teachers, special education teachers, and those with more experience in teaching students with special educational needs will report more positive attitudes towards inclusion.

2. Teachers from general education schools will report higher self-efficacy and more positive attitudes toward inclusion compared to teachers from special education schools.

3.a. Among teachers from general education schools, a positive association will be found between knowledge of inclusion policy, school support and self-efficacy as well as attitudes towards inclusion.

3.b. Among teachers from general education schools, a positive association will be found between teacher self-efficacy and their attitudes towards inclusion.

4. Among teachers from general education schools, self-efficacy will mediate the relationship between knowledge of inclusion policy, school support, and attitudes toward inclusion.

Materials and Methods

Participants

Participants were 352 teachers, mostly female (91.6%), who worked either in general (n=252) or special (n=100) education schools. More respondents (58%) had training in general education and 42% were special educators. Teachers' mean age was 41.5 (SD=10.48); 61.0% reported having either a BA or BEd degree and 39.0%

reported having an M.A. degree. Teachers had a mean of 15.0 years of experience (SD = 10.15), and 85.8% had taught students with special educational needs in the past. Only 21.6% of teachers reported receiving some type of training regarding inclusion. Finally, 60.6% were homeroom teachers and 39.4% were content area teachers.

Instruments

Teachers completed the following measures:

Attitudes towards inclusion were measured using two scales. Eighteen items were taken from the Multidimensional Attitudes Toward Inclusive Education Scale [MATIES; 3]. For example, "I believe an inclusive school is one that permits academic progress of all students regardless of their ability." Items were rated on a 5-point scale ranging from 1 ("disagree very strongly") to 5 ("very strongly agree"). The scale has three factors (each having 6 items): cognitive, affective and behavioral aspects of attitudes. Internal consistency was reported as between α=.77 to α=.91 in previous studies [3], and α=.76, α=.81, and α=.89 in the current study. A mean score was calculated for each of the three factors by averaging its items.

Twelve items were taken from the Teacher's Attitudes Toward Inclusion Scale [TATIS; 4]. Two items from the original scale were removed, as they were similar to items in the MATIES. In all items, the term "mild to moderate disability" was changed to "moderate to severe disabilities" as we suspected that it would be easier for teachers to report on positive attitudes when it comes to students with less complex disabilities. For example, "It is seldom necessary to remove students with moderate to severe disabilities from regular classrooms in order to meet their educational needs." Items were rated on a 5-point scale ranging from 1 ("disagree") very strongly to 5 ("very strongly agree"). The scale has three factors based on mean

scores: Teacher perception of students with moderate to severe disabilities in inclusive settings (4 items, α = .80), beliefs about the efficacy of inclusion (4 items, α = .86), and perceptions of professional roles and functions (4 items, α = .68). Reliabilities in the current study were α = .76, α = .74 and α =.70 for each of the subscales, respectively.

Teacher's sense of self-efficacy was measured using the 12-item Teacher's Sense of Efficacy Scale [TSES, 40] which measures teacher evaluations of their own likely success in teaching children with disabilities. Participants were asked to rate to what extent they are able to achieve each of the item statements. For example, "How much are you able to control disruptive behavior in the classroom?" Items were rated on a 5-point scale ranging from 1 ("not at all") to 5 ("to a great extent"). The scale has three factors as well as an overall score. We used the overall score based on its high reliability in previous studies [α = .90; 40] and in the current study (α =.91).

Subjective knowledge about inclusion policy was measured by one item developed for this study: "To what extent do you feel you have knowledge on the principles of the current Ministry of Education's policy about inclusion?" ranked on a 5-point scale from 1 ("very little") to 5 ("very much").

Perception of school support for inclusion: Teachers in general education schools were asked to answer four items developed specifically for this study: To what extent do you feel that: (1) you receive support from your school in dealing with students who are included? (2) the educational staff in your school is minded towards inclusion? (3) your school principal is focused on inclusion?, and (4) you are satisfied from the inclusion program in their school? Each item was rated on a 5-point scale ranging from 1 ("very little") to 5 ("very much"). A mean score was calculated for all four items with reliability of α = .91

Demographic and work-related variables: These included gender, age, teacher role (homeroom or content area teacher), academic degree, years of teaching experience, training (general or special education), experience in working with students with special educational needs (yes or no), training in inclusion (binary), type of school (binary: general or special education).

Procedures

The study protocol was approved by the (name concealed) University's IRB. Teachers were recruited from a random sample of 43 schools in Israel, from among 61 schools that were located in the cities that were first to implement the current Ministry of Education's policy about inclusion (before implementation was to take place across the country). All teachers within the sampled schools were asked to complete the questionnaires; however, in adherence to ethical guidelines, they were not mandated to respond. Most questionnaires were completed by the teachers when a research assistant visited the school while some completed an online version of the questionnaire. Data for this study were collected as part of a Ministry of Education sponsored study of educational reforms led by the research team.

Analyses

Means and standard deviations were used to examine the central tendency of the main study variables. For hypothesis 1, Pearson correlations and *t*-tests were calculated to assess the relationships between teachers' self-efficacy and attitudes toward inclusion and the background variables. For hypothesis 2, multivariate analyses of covariance were calculated to examine the differences in teacher self-efficacy and attitudes toward inclusion, between teachers in general education and teachers in special education. For hypothesis 3, multiple hierarchical regressions were calculated for teachers within the general education system, to assess: a) the

association between background variables, knowledge, and school support of inclusion and teachers' self-efficacy, and b) the relationships between background variables, knowledge, school support of inclusion, teachers' self-efficacy and teachers' attitudes toward inclusion. For hypothesis 4, the PROCESS procedure [41] was used to assess the mediating role of teachers' self-efficacy between knowledge about inclusion and school support of inclusion, and teachers' attitudes toward inclusion. Prior to data analysis, we analyzed missing data based on Little's test of Missing Completely at Random (MCAR). Results were non-significant, meaning that there missing data were random and so pairwise deletion was appropriate.

Results

Descriptive statistics

Table 1 presents the means and standard deviations of the components of teachers' attitudes towards inclusion and teachers' self-efficacy for the overall sample. As indicated, relatively strong positive attitudes were reported on the behavioral scale and for perception of teachers' professional roles and functions, while moderately positive attitudes were expressed on the affective and cognitive scales. Less positive attitudes were found regarding teachers' beliefs about the efficacy of inclusion and attitudes below the midpoint of the scale were reported for teachers' perception of students with disabilities within inclusive settings. Overall, most teachers reported high levels of self-efficacy.

Table 1. Means and standard deviations of main study variables (N=352)

	Range	Mean	SD
**Attitudes towards inclusive education**			
MATIES			
Cognitive	1.33-5.00	3.39	0.76
Affective	1.00-5.00	3.57	0.85
Behavioral	1.00-5.00	3.91	0.78
TATIS			
Perception of students with disabilities	1.00-5.00	2.25	0.83
Beliefs about efficacy of inclusion	1.00-5.00	2.64	0.86
Perception of professional roles and functions	1.00-5.00	3.78	0.75
**Teachers' self-efficacy**	2.00-5.00	4.12	0.53

Note: All scales range from 1 to 5 with higher ratings representing more positive attitudes.

Associations between background variables and attitudes toward inclusion

We first examined the relationship between demographic and occupational variables and dimensions of teachers’ attitudes toward inclusion and self-efficacy (hypothesis 1). Significance levels was set at .007 based on Bonferoni corrections for seven comparisons for each background variable.

Results showed that compared to teachers who were not trained to work with children with special education needs, teachers who were trained were more positive in the TATIS subscale perception of students with moderate to severe disabilities ($M = 2.66$, $SD = 0.81$ vs. $M = 2.14$, $SD = 0.80$; $t(341) = 4.89$, $p < .001$, $d = .53$); more

positive in the MATIES cognitive component of attitudes ($M = 3.69$, $SD = 0.70$ vs. $M = 3.31$, $SD = 0.75$; $t(341) = 3.82$, $p < .001$, $d = .41$); and more positive in the MATIES behavioral component of attitudes ($M = 4.13$, $SD = 0.75$ vs. $M = 3.84$, $SD = 0.78$; $t(341) = 2.79$, $p = .006$, $d = .30$). Years of teaching experience was negatively correlated with the MATIES affective component of attitudes towards inclusion ($r = -.15$, $p = .005$), suggesting that the higher the teaching experience, the lower the affective aspect of attitude toward inclusion. Teacher self-efficacy beliefs were moderately higher for homeroom teachers ($M = 4.19$, $SD = 0.48$) than for content area teachers ($M = 4.00$ $SD = 0.59$; $t(245.08) = 3.11$, $p = .002$, , $d = .40$); and slightly higher for teachers trained to work with children with special educational needs ($M = 4.28$, $SD = 0.44$) than for teachers without such training ($M = 4.06$, $SD = 0.54$; $t(341) = 3.34$, $p < .001$, $d = .36$).

Next, we examined the relationship between demographic and occupational variables and dimensions of teachers’ attitudes toward inclusion and self-efficacy only among teachers who worked in general education settings. Compared to teachers who were not trained to work with children with special educational needs, teachers who were trained to work with children with special educational needs reported more positive TATIS perception of students with disabilities ($M = 2.68$, $SD = 0.83$ vs. $M = 2.24$, $SD = 0.83$; $t(243) = 3.81$, $p < .001$, $d = .49$) and higher on the MATIES cognitive component of attitudes ($M = 3.71$, $SD = 0.70$ vs. $M = 3.47$, $SD = 0.67$; $t(243) = 2.47$, $p = .014$, $d = .32$). Years of teaching experience was negatively correlated with the MATIES affective component of attitudes ($r = -.19$, $p = .003$). Further, among teachers who worked in general education settings, reported self-efficacy was moderately higher for teachers trained to work with children with special educational needs ($M = 4.29$, $SD = 0.44$) compared with those without such training

(*M* = 4.06, *SD* = 0.52; *t*(243) = 3.25, *p* = .001, *d* =.42). Other demographic and background variables were unrelated with teacher self-efficacy or attitudes toward inclusion. Thus, subsequent analyses controlled for teacher's role, teacher training, and years of teaching experience.

Differences between teachers in general and special education schools

While these findings indicate that teachers trained to work with children with special education needs expressed more positive attitudes, teachers in the Israeli educational system with and without such training may work in special or general education schools. Attitudes toward inclusion and teachers' self-efficacy were, therefore, compared between teachers in general education and special education settings (hypothesis 2). Multivariate one-way analyses of covariance for the three TATIS subscales (teacher perceptions, beliefs about the efficacy of inclusion, and perceptions regarding professional roles) by school type, with teacher's role (homeroom teacher=1 vs. professional teacher=0), teacher training (trained to work with children with special educational needs=1 vs. not trained=0), and years of teaching experience as covariates.

The analysis revealed a main effect for the three TATIS scales, Wilks Λ = 0.95, $F_{(3, 325)} = 6.10$, $p < .001$, $\eta^2 = .05$. Among the covariates, only teacher training [Wilks Λ = 0.97, $F_{(3, 325)} = 3.93$, $p < .01$, $\eta^2 = .04$] and teaching seniority [Wilks Λ = 0.97, $F_{(3, 325)} = 3.12$, $p < .05$, $\eta^2 = .03$] were significant. Follow-up tests of between subject effects only for school type, showed differences, but with small to moderate effect sizes, only for the teacher perception scale [$F_{(1, 327)} = 7.56$, $p < .05$, $\eta_p^2 = .02$] and the teacher belief scale [$F_{(1, 327)} = 14.76$, $p < .001$, $\eta_p^2 = .04$].

A similar MANCOVA analysis was performed based on the three MATIES scales (cognitive, affective, and behavioral) using the same covariates. The omnibus

test revealed a main effect for school type [Wilks Λ = 0.84, $F_{(3, 325)}$, $p < .001$, $\eta^2 = .16$] along with significant main effects for the teacher training [Wilks Λ = 0.98, $F_{(3, 325)}$, $p < .05$, $\eta^2 = .03$] and teaching seniority [Wilks Λ = 0.96, $F_{(3, 325)} = 4.70$, $p < .01$, $\eta^2 = .04$] covariates. Follow-up analyses showed significant differences only for the cognitive [$F_{(1, 327)} = 21.80$, $p < .001$, $\eta_p^2 = .06$] and the affective subscales [$F_{(1, 327)} = 9.59$, $p < .01$, $\eta_p^2 = .03$].

Table 2 shows that perception of students with moderate to severe disabilities in inclusive settings, along with beliefs about the efficacy of inclusion were more positive among teachers in general education than among teachers in special education settings. Cognitive component of attitudes toward inclusion were more positive among teachers in general education than in special education, while the opposite trend is evident concerning the affective component of attitudes.

Table 2. Means, standard deviations and *F* values for teachers' attitudes toward inclusion and self-efficacy (*N* = 332)

	**General education *M* (*SD*) (n = 235)**	**Special education *M* (*SD*) (n = 97)**	***F*(1, 327) (η^2)**
TATIS			
Perception of students with moderate to severe disabilities in inclusive settings	2.34 (0.84)	1.97 (0.71)	7.58** (.02)
Beliefs about the efficacy of inclusion	2.74 (0.84)	2.31 (0.80)	14.75*** (.04)

Perceptions of professional roles and functions	3.80 (0.76)	3.72 (0.73)	0.28 (.00)
MATIES			
Cognitive	3.52 (0.68)	3.03 (0.81)	21.80*** (.06)
Affective	3.48 (0.83)	3.81 (0.86)	9.59** (.03)
Behavioral	3.94 (0.75)	3.78 (0.85)	1.75 (.01)
Teachers' self-efficacy	4.13 (0.51)	4.06 (0.58)	0.12 (.00)

**$p < .01$, ***$p < .001$ Note: All scales range from 1 to 5.

Relationships between background variables, subjective knowledge of inclusion policy, school support of inclusion and teachers' self-efficacy and attitudes toward inclusion

As indicated above, knowledge of inclusion policy and perception of school support were examined only among teachers working in the general education system. Mean subjective knowledge for these teachers was 2.21 (SD=1.10) and mean school support of inclusion was 3.76 (SD=0.88). Two sets of multiple hierarchical regressions were calculated. In the first set, teacher self-efficacy was examined as the dependent variable. In the second set, teachers' attitudes towards inclusion was the dependent variable with each attitude factor examined separately. For the regression predicting self-efficacy, independent variables were knowledge of inclusion policy and school support. For the regression predicting each attitude factor, independent

variables were knowledge of inclusion policy, school support, and teachers' self-efficacy. As before, we controlled for the teacher's role (homeroom teacher=1 vs. professional teacher=0), teacher training (trained to work with children with special needs=1 vs. not trained=0), and years of teaching experience. In all regressions, control variables were entered in the first step, knowledge of inclusion policy and school support of inclusion were entered in the second step and teachers' self-efficacy was entered in the third step (Table 3).

Table 3. Multiple regressions for teacher self-efficacy and attitudes toward inclusion in the general education system (*N* = 235)

		TATIS			MATIES		
	Teacher self efficacy	Perc. of students	Beliefs	Perc. of role and function	Cognitive	Affective	Behavioral
	B	β	β	β	β	β	B
**Step 1**							
Teachers' role[1]	.15*	-.04	-.03	.05	-.01	.03	-.07
Training	.21**	.20***	.05	.08	.16*	.04	.19**
Years of Experience	-.02	.10	.10	-.12	-.02	-.17**	-.10
Adj.R^2	.05**	.04**	.001	.01	.01	.02	.03*
**Step 2**							
Teachers' role[1]	.09	-.08	-.06	.03	-.05	.01	-.11
Training	.14*	.14*	-.01	.05	.11	-.02	.13*
Years of Experience	-.02	.11	-.08	-.11	-.01	-.16*	-.08
Knowledge	.28***	.14*	.04	.07	.07	.07	.08
School support	.17**	.24***	.32***	.11	.28***	.27***	.31***
ΔAdj.R^2	.11***	.08***	.09***	.01	.08***	.07***	.11***
**Step 3**							
Teachers' role[1]	--	-.09	-.07	.01	-.07	-.02	-.11
Training	--	.13*	-.02	.02	.08	-.05	.09
Years of Experience	--	.11	-.08	-.11	-.01	-.16**	-.08
Knowledge	--	.11	.02	.01	.02	.01	.01
School support	--	.22***	.30***	.07	.25***	.23***	.27***
Self-efficacy	--	.10	.08	.23**	.21**	.23***	.27***
ΔAdj.R^2	--	.01	.001	.04**	.04**	.04***	.05***
Adj.R^2	.16	.13	.09	.06	.13	.13	.19
F (6, 228)	10.23***	6.65***	5.07***	3.43**	6.64***	6.90***	10.20***

*$p < .05$, **$p < .01$, ***$p < .001$ For self-efficacy df = 5, 229.

[1] Teachers' role = homeroom teacher versus professional teacher.

Results show that all regression models were significant, although rates of explained variance were quite low. Teachers' self-efficacy was predicted by their training, knowledge of inclusion policy, and school support. Unsurprisingly, teachers who were trained to work with children with special educational needs, had more knowledge of inclusion policy, and who perceived their schools to be more supportive of inclusion, reported higher levels of self-efficacy regarding inclusion.

Interestingly, teachers’ perceptions of students with moderate to severe disabilities in inclusive settings were predicted by training and school support, such that teachers who were trained to work with children with special educational needs, and who perceived their schools to be more supportive of inclusion, reported more positive perceptions of students with disabilities in inclusive settings.

Teachers’ beliefs about the efficacy of inclusion were explained only by school support. In other words, teachers who perceived their schools to be more supportive of inclusion reported more positive beliefs about its efficacy. Teachers’ perceptions of professional roles and functions were significantly explained only by their self-efficacy in the context of inclusion, so that teachers with high self-efficacy reported more positive perceptions of professional roles and functions regarding inclusion.

Cognitive, affective, and behavioral attitudes toward inclusion were primarily explained by school support of inclusion and teacher self-efficacy regarding their abilities to engage in inclusionary practices. Teachers who perceived their schools to be more supportive of inclusion, and who had higher self-efficacy, reported more

positive cognitive, affective, and behavioral attitudes toward inclusion. In addition, teachers with less experience in teaching reported more positive affective attitudes toward inclusion (hypothesis 3).

These results reveal that teacher self-efficacy regarding inclusion may mediate the relationships between knowledge of inclusion policy and school support, and several of the attitudes towards inclusion factors (namely, teachers' perceptions of professional roles and functions, cognitive, affective, and behavioral attitudes). These possible mediations, described in hypothesis 4, were examined using the PROCESS analysis [41]. As before, the teacher role and teacher training were controlled for.

Results presented in Table 4 show that all mediation models were significant, as all confidence intervals for the indirect effects were positive and all Z values were significant. Further, all mediation models reveal the same pattern. Higher perceived knowledge of inclusion policy was related to higher self-efficacy regarding inclusion, which, in turn, associated with more positive attitudes about inclusion. Similarly, higher perceived school support of inclusion was related to higher self-efficacy regarding inclusion, which was related with more positive attitudes about inclusion.

Table 4. Path coefficients and indirect effects for self-efficacy as a mediator between knowledge and school support and teachers' attitudes toward inclusion (*N* = 235)

Dependent Variable (DV)	Variable	Path Coefficients		Indirect effects		
		to DV Estimate (SE)	to Mediator Estimate (SE)	Estimate (SE)	Z (d)	95% CI
**TATIS**						
Perc. of role and function	Knowledge	.013 (.054)	.312 (.064)***	.057 (.018)	3.103** (0.413)	.024, .095
	Self-efficacy	.184 (.053)***				
Perc. of role and function	School support of inclusion	.055 (.049)	.210 (.062)***	.037 (.015)	2.480* (0.328)	.011, .068
	Self-efficacy	.175 (.051)***				
**MATIES**						
Cognitive attitudes	Knowledge	.029 (.047)	.312 (.064)***	.054 (.017)	3.158** (0.421)	.023, .091
	Self-efficacy	.171 (.047)***				
Cognitive attitudes	School support of inclusion	.180 (.042)***	.210 (.062)***	.029 (.012)	2.387* (0.315)	.008, .056
	Self-efficacy	.141 (.044)**				
Affective attitudes	Knowledge	.022 (.058)	.312 (.064)***	.071 (.022)	3.194** (0.426)	.030, .117
	Self-efficacy	.228 (.056)***				
Affective attitudes	School support of inclusion	.176 (.052)***	.210 (.062)***	.038 (.016)	2.311* (0.305)	.010, .075
	Self-efficacy	.181 (.054)***				
Behavioral attitudes	Knowledge	.021 (.049)	.312 (.064)***	.074 (.021)	3.526*** (0.473)	.036, .118
	Self-efficacy	.229 (.048)***				
Behavioral attitudes	School support of inclusion	.159 (.046)***	.210 (.062)***	.035 (.015)	2.305* (0.304)	.008, .069
	Self-efficacy	.169 (.048)***				

$^*p < .05$, $^{**}p < .01$, $^{***}p < .001$

Discussion

This study had two broad aims: First, to examine the association between self-efficacy and attitudes towards inclusion among Israeli elementary school teachers in general and special education schools. Second, among teachers working in general education schools, to examine the role of teacher self-efficacy as a mediating variable between knowledge of inclusion policy, perception of school support, and teacher attitudes towards inclusion.

Results of the current study indicate that teachers rate themselves highly on self-efficacy regarding their perceived ability to teach students with disabilities. While this is encouraging, other findings from our study are cause for concern. First, though self-efficacy was associated with training, only 21% of teachers in our study reported receiving any sort of training on inclusion. Second, teachers rated their attitudes towards students with disabilities in inclusive settings and their beliefs about the efficacy of inclusion as relatively low. Third, although their perception of school support regarding inclusion was moderate, teachers perceived their subjective knowledge of the relevant inclusion policy as quite low. Taken together, these findings raise concerns regarding the state of inclusive education in Israel. This will be expanded upon below in discussing the results of our mediation models.

A unique contribution of the current study is its focus on teachers in both special and general education settings. We argue that attitudes of teachers in both these settings are of importance as each plays a role in the success of an inclusive education system. While the attitudes reported by teachers in both these settings were only moderate, those of teachers within the general education system were relatively higher on most scales. These results are somewhat different than those of previous

studies that reported on more positive attitudes among special education teachers, albeit most these studies were conducted in general education settings [5, 13 ,14].

On the one hand, these findings are encouraging given that teachers who work within general education settings are the main agents in implementing inclusion [13]. On the other hand, these findings are also a cause for concern, since we posit that teachers in special education settings must be aware of the ideological basis and practical significance of inclusion in order to make appropriate recommendations regarding transferring students with special educational needs into general settings. As we have stated elsewhere, there can be no policy without a supportive ideology, and no praxis without supportive policy [42].

Discussion of mediation models: Knowledge of inclusion policy, school support of inclusion, and self-efficacy

Findings from our mediation models show that while knowledge of inclusion policy was not directly associated with teachers' attitudes towards inclusion, indirect associations were significant via self-efficacy. Specifically, greater familiarity with inclusion policy was associated with greater self-efficacy and the latter was associated with teacher perception of their professional roles and functions, cognitive, affective and behavioral attitudes.

Somewhat differently from previous studies [e.g. 23, 24, 25], our study examined knowledge of inclusion policy rather than knowledge regarding the practice of inclusion itself. Similar to those previous studies, however, our findings highlight the importance of knowledge and our results point to the importance of being knowledgeable in local and national policy as a vital step towards greater feelings of self-efficacy in being able to implement effective educational practice. This is particularly important since, as suggested by other studies [17] and our own results,

teachers with increased self-efficacy, are more likely to have positive attitudes towards inclusion.

Based on the current findings, it is clear that in order to enhance inclusion in the Israeli educational system, local and national policy must be clearly communicated to teachers. Teachers must become knowledgeable in the current policy, its ideological basis, and means by which is should be implemented. In keeping with the 3P policy model (Philosophy, Policy, and Praxis) [42], teachers must understand the philosophy and ideology behind the policy before being asked to take an active role in implementing it [42].

Our findings also indicate that school support of inclusion had both a direct and an indirect association with teachers' attitudes towards inclusion. Specifically, higher levels of school support regarding inclusion were associated with greater self-efficacy, while the latter was associated with teachers' perception of their professional roles and functions, as well as cognitive, affective and behavioral attitudes. Greater school support of inclusion was also directly associated with cognitive, affective and behavioral attitude factors.

These findings provide strong support for the importance of leadership within schools [17, 28]. Working in a positive and supportive school environment may provide teachers with enhanced feelings of resilience regarding their capacity to teach students with disabilities [17]. This, in turn, may lead to more positive teacher attitudes towards the inclusion of these children. These findings also highlight the importance of provision of advanced training to teachers at all levels. Specifically, all teachers, including homeroom teachers, content area teachers and special education teachers should be provided with training in inclusive education. This training should

be provided beginning with the earliest stages of their careers and should continue on an ongoing basis.

Study limitations and future research:

The strength of the current study is in its examination of teachers in both special and general education settings and in the collection of data from a large sample of schools across the country. One limitation is its focus on attitudes towards inclusion rather than examining the actual practice of teachers working with students with disabilities. Additionally, this study is based on self-reports which may be susceptible to social-desirability bias. Since our study was anonymous we hope this did not occur. If it did, this would indicate that actual attitudes might be even lower than those reported.

Future studies should focus on actual practice implemented by teachers in various school settings. This could potentially be achieved by self-reports, but would best be measured via observational protocols within class time. Further, studies should focus on the effects of inclusion on students with disabilities, focusing on student academic and social achievements. One possible avenue for conducting such studies is by utilizing student self-report of their experiences in their class and school.

Conclusions and Implications

Findings from this study highlight the importance of knowledge of inclusion policy, school support of inclusion, teacher's self-efficacy, training, and working in general education settings as being important predictors of teachers' attitudes towards inclusion. In terms of implications regarding the advancement of the field of inclusive education, teacher training programs should not distinguish between the training of special education and general education teachers [42]. This would send a message that

all teachers, regardless of their workplace must be capable of teaching heterogeneous classrooms and knowledgeable regarding inclusionary practices.

Acknowledgments:

This study was conducted with the help of a grant provided by the Ministry of Education. We would like to thank all research assistants who helped in carrying out this study. Special thanks to Tziva Elgart for her assistance.

References

1. UNESCO International Bureau of Education. Defining an inclusive education agenda: Reflections around the 48th session of the international conference on education. UNESCO digital library. 2009;1-115. Available from: http://www.ibe.unesco.org
2. Murphy K. Teacher Attitudes toward inclusion practices [dissertation]. Mount Saint Vincent University; 2014. Available from: http://dc.msvu.ca
3. Mahat M. The development of a psychometrically-sound instrument to measure teachers' multidimensional attitudes toward inclusive Education. Int J Spec Educ. 2008; 23(1): 82-92. Available from: https://eric.ed.gov/?id=ej814377
4. Cullen JP, Gregory JL, Noto LA. The teacher attitudes toward inclusion scale (TATIS) technical report. Online submission. 2010 FEB 11. Available from: https://files.eric.ed.gov/fulltext/ED509930.pdf.
5. Charley CY. General education and special education teachers' attitudes toward inclusion [dissertation]. Walden University. 2015. Available from: https://scholarworks.waldenu.edu/cgi/viewcontent.cgi?referer=https://scholar.google.com/&httpsredir=1&article=1457&context=dissertations
6. Saloviita T. Attitudes of teachers towards inclusive education in Finland. Scandinavian Journal of Educational Research. 2020;64(2):270-282. doi: 10.1080/00313831.2018.1541819.
7. Goddard C, Evans D. Primary pre-service teachers' attitudes towards inclusion across the training years. Australian Journal of Teacher Education. 2018;43(6):122-142. http://dx.doi.org/10.14221/ajte.2018v43n6.8.
8. Moberg S, Muta E, Korenaga K, Kuorelahti M, Savolainen H. Struggling for inclusive education in Japan and Finland: teachers' attitudes towards inclusive

education. Eur J Spec Needs Educ. 2020;35(1): 100-114. https://doi.org/10.1080/08856257.2019.1615800

9. Takala M, Pirttimaa R, Törmänen M. Inclusive special education: The role of special education teachers in Finland. Brit. J. Educ. Psychol. 2009;36(3):162-172. https://doi.org/10.1111/j.1467-8578.2009.00432.x.
10. Saloviita T. Teacher attitudes towards the inclusion of students with support needs. J Res Spec Educ Needs. 2020;20(1):64-73. https://doi.org/10.1111/1471-3802.12466.
11. Malki S, Einat T. To include or not to include - this is the question: Attitudes of inclusive teachers toward the inclusion of pupils with intellectual disabilities in elementary schools. Educ Citizsh Soc Justice. 2018;13(1):65-80. https://doi.org/10.1177/1746197917705138.
12. Horne PE, Timmons V. Making it work: Teachers' perspectives on inclusion. International journal of inclusive education. 2009;13(3):26-41. https://doi.org/10.1080/13603110701433964
13. Avramidis E, Toulia A, Tsihouridis C, Strogilos V. Teachers' attitudes towards inclusion and their self- efficacy for inclusive practices as predictors of willingness to implement peer tutoring. J Res Spec Educ Needs. 2019;19:49-59. https://doi.org/10.1111/1471-3802.12477
14. Parker S. A comparison of the attitudes of secondary regular education and special education teachers toward inclusion of students with mild disabilities in their classrooms [dissertation]. Regent University. 2009. Available from: https://search.proquest.com/openview.
15. Bandura A. Self-efficacy: toward a unifying theory of behavioral change. Psychol Rev. 1997;84(2):191-215. https://doi.org/10.1037/0033-295X.84.2.191

16. Sharma U, Loreman T, Forlin C. Measuring teacher efficacy to implement inclusive practices. J Res Spec Educ Needs. 2012;12(1);12–21. https://doi.org/10.1111/j.1471-3802.2011.01200.x

17. Wilson C, Marks-Woolfson L, Durkin K. School environment and mastery experience as predictors of teachers' self-efficacy beliefs towards inclusive teaching. International journal of inclusive education. 2020;24(2):218-234. https://doi.org/10.1080/13603116.2018.1455901

18. Brady K, Woolfson L. What teacher factors influence their attributions for children's difficulties in learning? Br J Educ Psychol. 2008;78(4):527-544. https://doi.org/10.1348/000709907X268570

19. Fisher Y. Predicting Teachers' Perception of Inclusion: What is the role of self-efficacy? EC Psychol Psychiatr. 2017; 2(5): 157-171. Available from: https://www.researchgate.net/profile/Yael_Fisher/publication/315194044_EC_PSYCHOLOGY_AND_PSYCHIATRY_Research_Article_Predicting_Teachers'_Perception_of_Inclusion_What_is_the_Role_of_Self-Efficacy/links/58cc0c4c4585157b6dabff19/EC-PSYCHOLOGY-AND-PSYCHIATRY-Research-Article-Predicting-Teachers-Perception-of-Inclusion-What-is-the-Role-of-Self-Efficacy.pdf

20. Savolainen H, Engelbrecht P, Nel M, Malinen O. Understanding teachers' attitudes and self-efficacy in inclusive education: Implications for pre-service and in-service teacher education. Eur J Spec Needs Educ. 2010;27(1);51-68. https://doi.org/10.1080/08856257.2011.613603

21. Savolainen H, Malinen OP, Schwab S. Teacher efficacy predicts teachers' attitudes towards inclusion–a longitudinal cross-lagged analysis. International journal of inclusive education. 2020 APR;1-15. Available from: https://doi.org/10.1080/13603116.2020.1752826

22. Malinen O, Savolainen H, Xu J. Beijing in-service teachers' self-efficacy and attitudes towards inclusive education. Teach Teach Educ. 2013;28(4):526-534. https://doi.org/10.1016/j.tate.2011.12.004

23. Gavish B. Four profiles of inclusive supportive teachers: Perceptions of their status and role in implementing inclusion of students with special needs in general classrooms. Teach Teach Educ. 2017;61:37-46. https://doi.org/10.1016/j.tate.2016.10.004

24. Idol L. Toward inclusion of special education students in general education: A program evaluation of eight schools. Remedial Spec Educ. 2006;27(2):77-94. doi:10.1177/07419325060270020601

25. Mangope B, Mannathoko MC, Kuyini AB. Preservice physical education teachers and inclusive education: Attitudes, concerns and perceived skills needs. Int J Spec Educ. 2013;28(3):82–92. https://files.eric.ed.gov/fulltext/EJ1024424.pdf

26. Forlin C, Chambers D. Teacher preparation for inclusive education: Increasing knowledge but raising concerns. Asia-Pacific Journal of Teacher Education. 2011;39(1):17-32. doi:10.1080/1359866X.2010.540850

27. Weatherley R, Lipsky M. Street-level bureaucrats and institutional innovation: Implementing special-education reform. Harv Educ Rev. 1977;47(2):171-197. https://doi.org/10.17763/haer.47.2.v870r1v16786270x

28. Ainscow M, Sandill A. Developing inclusive education systems: the role of organisational cultures and leadership. International journal of inclusive education. 2010;14(4):401-416. https://doi.org/10.1080/13603110802504903

29. Woodcock S, Woolfson LM. Are leaders leading the way with inclusion? Teachers' perceptions of systemic support and barriers towards inclusion. Int J Educ Res. 2019;93,232-242. https://doi.org/10.1016/j.ijer.2018.11.004

30. De-Boer A, Pijl SJ, Minnaert A. Regular primary school teachers' attitudes towards inclusive education: A review of the literature. International journal of inclusive education. 2011;15(3):331-353. https://doi.org/10.1080/13603110903030089

31. Vaz S, Wilson N, Falkmer M, Sim A, Scott M, Cordier R, et al. Factors associated with primary school teachers' attitudes towards the inclusion of students with disabilities. PLoS ONE. 2015;10(8). Available from: https://doi.org/10.1371/journal.pone.0137002

32. Leonard NM, Smyth S. Does training matter? Exploring teachers' attitudes towards the inclusion of children with autism spectrum disorder in mainstream education in Ireland. International journal of inclusive education. 2020;1-15. https://doi.org/10.1080/13603116.2020.1718221

33. Yadav M, Das AJ, Sharma S, Tiwari A. Understanding teachers' concerns about inclusive education. Asia-Pacific Education Review. 2015;16(4):653-662. https://doi.org/10.1007/s12564-015-9405-6

34. Chhabra S, Srivastava R, Srivastava I. Inclusive education in Botswana: the perceptions of school teachers. J Disabil Policy Stud. 2010;20:219-228. https://doi.org/10.1177/1044207309344690

35. Ahmmed M, Sharma U, Deppeler J. Variables affecting teachers' intentions to include students with disabilities in regular primary schools in Bangladesh. Disabil Soc. 2014;29(2),317-331. https://doi.org/10.1080/09687599.2013.796878

36. MacFarlane K, Marks-Woolfson L. Teacher attitudes and behavior toward the inclusion of children with social, emotional, and behavioral difficulties in mainstream schools: An application of the theory of planned behavior. Teach Teach Educ. 2013;29:46-52. https://doi.org/10.1016/j.tate.2012.08.006

37. Kurniawati F, Minnaert A, Mangunsong F, Ahmed W. Empirical study on primary school teachers' attitudes towards inclusive education in Jakarta, Indonesia. Procedia Soc Behav Sci. 2012;69:1430-1436. https://doi.org/10.1016/j.sbspro.2012.12.082

38. Huang C, Chen R. Attitudes toward inclusive education: A comparison of general and special education teachers in Taiwan. Asian Journal of Inclusive Education. 2017;5(1):23-37. Available from: https://www.researchgate.net/profile/Roy_Chen20/publication/Attitudes_toward_Inclusive_Education_A_Comparison_of_General_and_Special_Education_Teachers_in_Taiwan/links/Attitudes-toward-Inclusive-Education-A-Comparison-of-General-and-Special-Education-Teachers-in-Taiwan.pdf

39. Varcoe L, Boyle C. Pre-service primary teachers' attitudes towards inclusive education. Educ Psychol. 2014;34(3),323-337. https://doi.org/10.1080/01443410.2013.785061

40. Tschannen-Moran, M., & Hoy, A. W. Teacher efficacy: Capturing an elusive construct. Teach Teach Educ. 2001;17(7):783-805. https://doi.org/10.1016/S0742-051X(01)00036-1

41. Hayes AF**.** Introduction to mediation, moderation, and conditional process analysis. 2nd ed. New York: The Guilford Press. 2018.

42. Gumple TP, Koller J, Weintraub N, Werner S, Wiesenthal V. The 3P model for creating sustainable educational reform: An epilogue to the special issue. Journal of educational administration. (forthcoming).
