# Supplementary material for: Can self-efficacy mediate between knowledge of policy, school support and teacher attitudes towards inclusive education?
Source: PLoS One. 2021 Sep 20;16(9):e0257657. doi: 10.1371/journal.pone.0257657 (PMC8452075; doi:10.1371/journal.pone.0257657)
Supplement: Attachment — Submitted filename: Comments to PONE manuscript.docx [file pone.0257657.s001.docx]

Dear Editor,

Thank you for giving me the opportunity to read manuscript “**Can self-efficacy mediate between knowledge of policy, school support and teachers' attitudes towards inclusive education?”**

It is indeed a new learning and insight about inclusive education. I congratulate the authors for taking up specially a less explored variable i.e. teacher’s knowledge about inclusive education policy.

While I found the paper quite relevant in today’s context, I also have some serious concerns in the manuscript as several points are ambiguous. I have highlighted in the manuscript with comments. I also mention them below:

1. The references are mentioned as numbers which makes it difficult to see the reference to make a connect, is this the way the journal asks to give references? since I have used most of the references in my work hence, I have a fair idea about the studies cited here.
2. I strongly recommend to strengthen the argument of relation between attitudes and knowledge about country inclusive policy. This can be done by giving specific information about Israel inclusive education policy and the gaps between reaching to “streets” as the authors mention, hence the need to measure it. In the current form it is loosely knit and sounds less convincing as there are lot of studies on attitudes and self efficacy.
3. The link can be: attitudes → self efficacy → knowledge about policy →need for training module . may be this is a part of a bigger study but in the current form it requires a firm argument.
4. I wonder what was the specific reason for picking up Self efficacy and knowledge about policy of education needs. Maybe it has a specific relevance to Israeli context but it has not been brought about. I wonder because there are loads of literature on teacher’s self-efficacy towards inclusion. Perhaps the authors need to make a connection in the specific country context. Moreover, there should be more information about the country specific inclusive education national policy for international readers.
5. The three aspects on which the manuscript focuses are : self efficacy, attitudes , knowledge about policy of inclusive education. Then why other variable such as school leadership , climate support system etc?
6. About the section which has mentioned aims of the study: how are 3a and 3b different? Why have authors decided to have two parts?
7. About the instrument: why were two scales chosen to measure the attitudes? When most of the items are overlapping? The MATIES is quite comprehensive and fits in the theoretical framework also. Only one item to measure knowledge about policy of inclusion? Moreover, is there any specific policy in Israel? what is the significance of teachers to know it? Generally, decisions to make school inclusive is taken by the school leaders or management not the teachers. Teachers play key role in implementing it.
8. About the instrument: details such as final items of the scale should be provided and the last part (see highlighted comments within the manuscript) seems incomplete.
9. The demographic information should be provided in a table which makes it easier to read.
10. Paper requires reorganization and the manuscript has potential to contribute in the research body. It requires a consistency in the use of terms to avoid confusions and make clear for international readers.
11. A suggestion for clear organization is as under:

The four aspects of inclusion in Israel

Inlcusive education

attitudes

self-efficacy

knowledge about policies
